# Estimating the economic burden of invasive non-typhoidal Salmonella infections in low- and middle-income countries

**DOI:** 10.1136/bmjgh-2025-019370

**Published:** 2025-11-08

**Authors:** Jung-Seok Lee, Yongha Hwang, Calman Alexander MacLennan, Mark Jit, Jean-Louis Excler, Jerome H Kim

**Affiliations:** 1 Policy and Economic Research, International Vaccine Institute, Seoul, Korea (the Republic of); 2 Enteric and Diarrheal Diseases, Bill and Melinda Gates Foundation, Seattle, Washington, USA; 3 Jenner Institute, Nuffield Department of Medicine, University of Oxford, Oxford, England, UK; 4 Department of Global and Environmental Health, New York University, New York, New York, USA; 5 Department of Infectious Disease Epidemiology, London School of Hygiene and Tropical Medicine, London, UK; 6 Business Development, International Vaccine Institute, Seoul, Korea (the Republic of); 7 International Vaccine Institute, Gwanak-gu, Korea (the Republic of)

**Keywords:** Health economics, Global Health, Health policy

## Abstract

**Introduction:**

Invasive non-typhoidal *Salmonella* (iNTS) disease is a global health concern, particularly for sub-Saharan Africa. Despite high case fatality risks, there is no vaccine available against the disease. An obstacle to vaccine development is a lack of data on the economic burden of iNTS disease in many parts of the world. The main aim of the current study is to estimate the economic burden of iNTS disease in 123 countries.

**Methods:**

Several multivariate regression models were parameterised with the data obtained from an existing systematic literature review on the economic burden of iNTS disease and all forms of NTS disease. Various model diagnostics were performed to validate the statistical significance of each model outcome, and the most suitable model was selected to predict costs in countries where no data points were available.

**Results:**

A generalised linear model with gamma distribution with log link was chosen based on model diagnostics. While the average economic burden per iNTS disease episode ranged from US$341 in Africa to US$2194 in Europe, the total economic burden of iNTS disease was the highest in Africa due to the high burden of the disease in the region.

**Conclusion:**

The current study indicates that the economic burden of iNTS disease is substantial. Given the scarcity of field-based iNTS disease economic burden estimates, cost extrapolation through an econometric framework can be helpful for understanding the associated cost implications in a resource-limited setting and for informing the cost-effectiveness of public health interventions including future vaccination strategies.

**WHAT IS ALREADY KNOWN ON THIS TOPIC**

- The economic burden of invasive non-typhoidal *Salmonella* (iNTS) is highly scarce according to a previous systematic literature review study. In particular, the number of existing studies is disproportionately lower in low- and middle-income countries (LMICs), which makes it difficult to evaluate the accurate societal impact of future vaccines in pipeline in LMICs where the disease is highly prevalent.

**WHAT THIS STUDY ADDS**

- The current study estimates the economic burden of iNTS disease in countries where no primary data points are available, through an econometric modelling framework. Four different types of econometric models are constructed to identify the most suitable model to explain the variance of existing cost data and further used to estimate the economic burden of iNTS disease in 123 countries.

**HOW THIS STUDY MIGHT AFFECT RESEARCH, PRACTICE OR POLICY**

- Considering the societal and economic impacts of vaccination are largely uncertain due to the lack of economic evaluation studies for iNTS disease, understanding the economic burden of this disease is an important step to make standardised comparisons among health problems competing for countries’ limited resources. The proposed cost estimation through the econometric framework helps estimate the robust economic burden of iNTS disease and assess the value of iNTS-containing vaccines.

## Introduction

 Invasive non-typhoidal *Salmonella* (iNTS) disease is caused by systemic bacterial infection with non-typhoidal serotypes of *Salmonella* (NTS), particularly *Salmonella Typhimurium* and *Salmonella Enteritidis*.^1^ The *Salmonella* genus comprises over 2500 serovars, and most of these can cause various diseases, including iNTS.^2 3^ The most vulnerable populations include children grappling with malnutrition, malaria and HIV infection, as well as immunocompromised adults with HIV.^1^ According to the Institute for Health Metrics and Evaluation estimation, the global disease burden of iNTS was estimated to be 510 000 cases, 62 000 deaths and 4.74 million global disability-adjusted life years (DALYs) in 2021.^4^ Most iNTS disease burden was concentrated in sub-Saharan Africa showing 52 096 deaths and 4.12 million DALYs due to the high prevalence of risk factors.

Despite the high mortality and morbidity of the disease, there are significant knowledge and research gaps in epidemiology and potential health impacts due to iNTS disease. For example, a better understanding is needed on reservoirs, sources, duration and prevalence of carriage, as well as the role of serotypes beyond *S*. *Typhimurium* and *S*. *Enteritidis* as the cause of invasive disease.^5^ In addition, the number of population-based surveillance studies is insufficient, making it difficult to generate reliable disease burden estimates at the country, regional and global level.

While there is no licensed vaccine against iNTS, a bivalent iNTS vaccine is currently in development at the GSK Vaccines Institute for Global Health targeting *S*. *Typhimurium* and *S*. *Enteritidis*, the two predominant NTS serovars causing bacterial bloodstream infections with high case fatality risks.^6^ In addition, there are other combination *Salmonella* vaccines in the pipeline, and one of the most advanced candidates is a trivalent conjugate vaccine which is based on an existing typhoid conjugate vaccine (TCV), Typbar-TCV (Bharat Biotech, India), and includes two NTS components (*S*. *Typhimurium* and *S*. *Enteritidis*).^6^ Given the absence of licensed vaccines against iNTS disease, there is a large degree of uncertainty about vaccine profiles including efficacy and duration of protection, as well as the economic impact of vaccination.^7 8^

According to a recent systematic literature review,^9^ few economic burden studies exist for iNTS disease and, indeed, all forms of NTS disease. In particular, the review indicated that field-based economic burden studies (primary data) for iNTS disease were particularly scarce in low- and middle-income countries (LMICs), compared with studies performed for diarrhoeagenic NTS disease in high-income countries. For example, existing studies on NTS disease in high-income countries reported that the estimated direct medical cost ranged from US$262 to US$8451 in North America (the USA, Canada) and from US$229 to US$6744 in Europe (UK, Sweden, Poland and the Netherlands).^9^ The absence of iNTS economic burden data at the global level hampers an accurate cost-effectiveness analysis which is critical for public health interventions including decisions concerning investment in developing vaccines and later implementation of vaccination programmes. Understanding the economic burden of this disease is an important step to make standardised comparisons among competing health problems, given limited resources.

Considering that many countries lack primary data on the economic burden of iNTS disease, the main aim of the current study lies in estimating the economic burden of iNTS disease in countries where no primary data points are available, through an econometric modelling framework. This approach is vital as it allows us to fill out existing knowledge gaps in the economic burden of iNTS, potentially shaping our understanding and response to the impact and cost-effectiveness of future iNTS vaccines.

## Methods

### Data preparation

Existing economic burden studies for iNTS and NTS disease were identified in the previous systematic literature review.^9^ Given heterogeneous methods adopted in the existing economic burden literature, the study outcomes were standardised as total costs reflecting direct medical costs (DMC), direct non-medical costs (DNMC) and indirect costs (IC) explicitly or implicitly. For the studies which did not report a DNMC or IC, any missing cost category was calculated based on the following approach: (1) if other studies from the same country existed, the same cost category estimated at a different time point was applied after inflating or deflating, (2) if no other studies were available from the same country, the average of the cost component among the countries in the same income group and/or region was adopted. To increase the number of data points, two unpublished field-based economic burden studies for iNTS disease carried out by the International Vaccine Institute (IVI) were additionally included. The number of data points per cost category prior to the imputation was 47, 15 and 21 for DMC, DNMC and IC, respectively. All cost data were inflated to the year 2021 based on the Gross Domestic Product (GDP) deflator obtained from the World Bank.^10^ A wide range of indicators which were used in existing literature^11^,^13^ or considered to be relevant in association with treatment costs were used as explanatory variables to explain the variation in the dependent variable (costs) by time (year) and location (country) as shown in table 1.^11 14 15^

**Table 1:** List of explanatory indicators

Indicator	Source
GDP per capita (current US$)	World Bank^26^
Population density (people per km^2^ of land area)	World Bank^27^
Urban population (% of total population)	World Bank^28^
Mortality rate, under-5 (per 1000 live births)	World Bank^29^
Hospital beds (per 1000 people)	World Bank^30^
Physicians (per 1000 people)	World Bank^31^
Cost per bed day by hospital level	WHO-CHOICE^32^
Cost per outpatient visit	WHO-CHOICE^32^
Life expectancy at birth, total (years)	World Bank^33^
Current health expenditure per capita (current US$)	World Bank^34^
Domestic general government health expenditure (% of general government expenditure)	World Bank^35^
WHO region	WHO^36^

GDP, Gross Domestic Product.

### Model specifications

Given that the nature of cost data is generally right-skewed with increasing variability, an ordinary least squares (OLS) model with a log-transformation of the dependent cost variable has been one of the common methods used in existing literature.^11 16 17^ While the logarithmic transformation of a cost dependent variable often makes a right skewed distribution symmetrical, the interpretation and inferences of the primary outcome (cost) are not always straightforward with an OLS log cost model. A simple back transformation (in this case, the exponent of the estimated outcome) does not provide the expected outcome because in OLS with a log transformation, the expectation before back-transforming is $E\left[ln(y)|x\right]$ instead of $lnE\left[y|x\right]$. For OLS with non-normally distributed homoscedastic errors, the smearing estimate has been widely used as a correction factor.^16 18^ With Duan’s smearing factor (ϕ), the predicted costs can be estimated as $E\left(y\right)=exp(x\beta )∙\phi$.

Other studies have adopted generalised linear models (GLMs) in the cost data analysis.^16 19^ Unlike OLS, GLMs link independent variables rather than the dependent variable, making the expectation to be $lnE\left[y|x\right]$ which removes the retransformation problem observed in OLS.^16^ Within the GLM framework, the gamma distribution with the log link function has been widely used, while the inverse Gaussian distribution with a high initial peak and a long right tail was also recommended for the analysis of healthcare costs.^16^ Thus, the current study compared the following multivariate regression models based on existing evidence and investigated the statistical validity of each to arrive at the most suitable model for the current cost data: (1) OLS, (2) OLS with the log-transformation of costs (log costs), (3) GLM with gamma distribution with log-link (gamma, log-link) and (4) GLM with inverse Gaussian distribution with log-link (inverse Gaussian, log-link).

### Model diagnostics

An initial set of the indicators shown in table 1 was first examined for statistical significance and Akaike information criteria (AIC) within the OLS framework: (1) normality of residuals, (2) multicollinearity, (3) heteroscedasticity and (4) AIC. While normality does not require unbiased estimates of coefficients, normality of residuals assures that the p values for hypothesis testing are valid. Thus, the quantiles of the residuals were plotted against the quantiles of a normal distribution to check sensitivity to non-normality. In addition, the Shapiro-Wilk W test was conducted to check normality. The variance inflation factor (VIF) was adopted to detect multicollinearity, as when the degree of multicollinearity increases, the estimated SEs of the coefficients can be inflated, rendering them unstable or insignificant. Given that high correlations among interaction terms, squared terms and main effects are normal and expected, the VIF was examined for the main effect terms. As a rule of thumb, a VIF value of 10 or greater was used to consider potential collinearity.^20^ The homogeneity of variance of the residuals (heteroscedasticity) was also examined using the Breusch-Pagan test, where the null hypothesis is that the variance of the residuals is homogeneous. If the variances of the residuals are not constant, then the variances of the residuals indicate a pattern against the fitted values. AIC was used to determine a superior model with a lower value of AIC.

With the final set of the indicators, the mean absolute error (MAE) and the root mean squared error (RMSE) were further measured and compared among all four multivariate regression models in order to identify the best-performing model.

### Outcome generation

Once the final model was selected after model validation, the economic burden per episode was estimated for iNTS disease with 95% CIs at the global level excluding high-income countries as classified by the World Bank.^21^ The CIs were generated based on the SEs of predictions that are non-linear functions of estimated model parameters using the delta method. The primary outcome was predicted based on the final set of indicators in 2021, and all values were expressed in US dollars using the official exchange rates as well as in international dollars based on the purchasing power parity.^10^ To understand the relative impact of iNTS disease economic burden by country, the proportion of the iNTS disease economic burden out of current health expenditure was further estimated by dividing the product of the economic burden including the productivity losses per iNTS disease episode and iNTS disease cases by the product of the current health expenditure per capita and the total number of populations for each country.

### Sensitivity analysis

Considering a degree of uncertainty due to data-sparse settings, the robustness of the model fit was further investigated by estimating varying variance-covariance matrix corresponding to the parameter estimates: (1) no adjustment, (2) Huber/white/sandwich estimator and (3) bootstrapping. The sandwich estimator assumes observations are independent but allows heteroskedasticity. Thus, this estimator is robust to heteroskedasticity. On the other hand, bootstrapping resamples the data with replacement to create multiple datasets, thus provides SEs that reflect the uncertainty associated with the resampling process. Given that the observations included in the current study were likely independent with suspected heteroskedasticity, the sandwich estimator was used as the default and compared with the other two methods to investigate the robustness of the model fit. Lastly, additional sensitivity analyses were carried out for the scenario where missing cost components were not imputed.

## Results

Among all economic burden studies identified in the previous systematic literature review,^9^ a total of 52 cost data points were eligible for the current study including the IVI’s own field-based economic burden studies. After conducting the series of model diagnostics within the OLS framework mentioned in the Methods, the following four covariates were selected as they met all four diagnostic criteria: GDP per capita (GC), life expectancy (LE), population density (PD), iNTS (dichotomous variable=1 if iNTS), interaction of GC and LE and squared PD^2^:


$$\begin{array}{l} y_{i}=\beta_{0}+\beta_{1}\cdot GC_{i}+\beta_{2}\cdot LE_{i}+\beta_{3}\cdot PD_{i}+\\ \;\beta_{4}\cdot iNTS_{i}+\beta_{5}\cdot GC_{i}LE_{i}+\beta_{6}\cdot PD_{i}^{2}+\varepsilon_{i} \end{array}$$

where, $y_{i}$ is the outcome variable (cost) and $\varepsilon_{i}$ is the random error term.

The mean VIF for the main effect terms was estimated to be 1.51. The Breusch-Pagan test indicated that the variance of the residuals was homogeneous for the OLS log costs model (p value of 0.498), whereas the null hypothesis of homogeneity of the variance of the residuals was rejected for the OLS model (p value of 0.000). Based on the Shapiro-Wilk W test, while the null hypothesis of a normal distribution was rejected for the OLS model, we failed to reject the null hypothesis for the OLS log costs model, meaning that the distribution is normal with the log-transformed costs.

**Figure 1:**
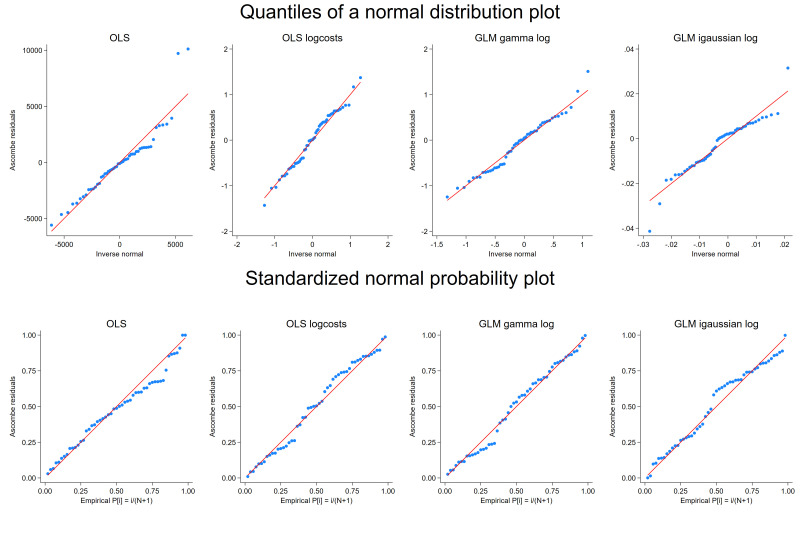
Quantile and standardised normal probability plot. The upper panel shows the quantiles of the variable against the quantiles of a normal distribution, while the lower panel shows the standardised normal probability plots for the four models. GLM, generalised linear model; OLS, ordinary least squares.

With the final set of the covariates, the model specification was further investigated with the GLM frameworks. In figure 1, the upper panel focuses on the sensitivity to non-normality near the tails, and the lower panel shows the sensitivity to non-normality in the middle range of the data. While the upper panel of figure 1 presents a deviation from normal at the upper tail in the OLS model, a weak indication of non-normality was observed in all four models in the standardised normal probability plot in the lower panel. The residuals are also plotted against the predicted values and presented in online supplemental figure 1. The variance of the residuals appeared to be relatively constant in the OLS log costs model and the GLM gamma log model, whereas the heteroscedasticity was observed in the OLS model (increase in the variance of the residuals) and the GLM inverse Gaussian log model (decrease in the variance of the residuals).

As shown in table 2, the normality (the Shapiro-Wilk W test) and the statistical significance of all coefficients were observed in the OLS log costs model and the GLM gamma log model. The lowest AIC value was estimated for the GLM gamma log model. It should be noted that while all the coefficients remained statistically significant for both OLS log costs and GLM gamma models with no adjustment, PD and squared PD were not significant at the significance level of 0.05 with the bootstrapped estimator (see online supplemental tables 2 and 3). In online supplemental table 1 where the model performance is compared in terms of MAE and RMSE, the GLM gamma log model produced the lowest MAE and RMSE, followed by the OLS log costs (smearing) and the OLS log costs (exponentiation). Combining all, the model diagnostic results indicated that the GLM gamma log model was the most suitable for the current cost data compared with the rest of the models and thus selected for the estimation of the economic burden per iNTS disease episode.

**Table 2:** Regression outputs (Huber/white estimator)

Variable	OLS			OLS with log costs			GLM (gamma, log)			GLM (inv. Gaussian, log)		
	Coefficients	SE	P value	Coefficients	SE	P value	Coefficients	SE	P value	Coefficients	SE	P value
Constant	−31 326.838	12 505.6	0.016	−9.458	1.6	0.000	−9.237	1.5	0.000	−8.609	0.9	0.000
GDP capita	1.735	0.8	0.032	0.001	0.0	0.000	0.001	0.0	0.000	0.001	0.0	0.000
LE	429.880	166.5	0.013	0.222	0.0	0.000	0.221	0.0	0.000	0.214	0.0	0.000
Pop. density	−7.915	2.3	0.001	−0.003	0.0	0.002	−0.003	0.0	0.000	−0.003	0.0	0.000
iNTS	5225.817	2747.9	0.064	0.768	0.3	0.005	0.670	0.2	0.006	0.413	0.2	0.062
GDP cap&LE	−0.021	0.0	0.034	0.000	0.0	0.000	0.000	0.0	0.000	0.000	0.0	0.000
Sq. density	0.001	0.0	0.001	0.000	0.0	0.002	0.000	0.0	0.000	0.000	0.0	0.000
AIC	973.1			Not comparable*			930.2			1302.9		
SW test (p value)	0.001			0.596			0.408			0.025		

* The AIC generated by fitting the log transformed outcome variable is not comparable to the rest of the models as the response variable is different.^37^

† See online supplemental tables 4-6 for the outcomes of the additional sensitivity analyses with no imputed cost assumption.

AIC, Akaike information criteria; GDP, Gross Domestic Product; GLM, generalised linear model; iNTS, invasive non-typhoidal *Salmonella*; LE, life expectancy; OLS, ordinary least squares; SW, Shapiro-Wilk W.

The estimated economic burden per iNTS disease episode is shown by country in online supplemental table 7 (see online supplemental table 8 for the estimated economic burden per NTS episode by country). Among 123 countries, the iNTS economic burden per episode was estimated to be highest in Thailand at US$5269 and lowest in Nigeria at US$18. The economic burden per iNTS disease episode was estimated to be lower for some of the upper- and middle-income countries (UMICs) in Africa such as Gabon, Equatorial Guinea, Botswana, South Africa and Namibia. This was mainly because their LE was lower than the respective income group average and combined with the relatively greater impact of the LE covariate compared with other independent covariates.

**Figure 2:**
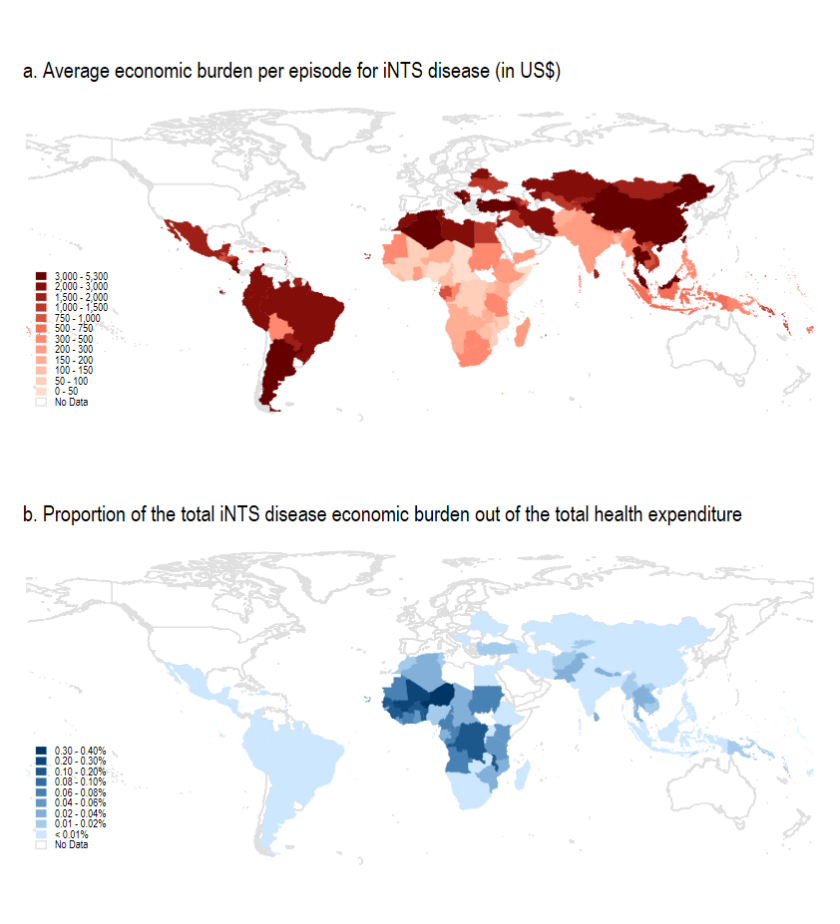
Economic burden of iNTS disease by country. The health expenditure indicator was not available in Libya, Somalia, Palestine and Yemen. iNTS, invasive non-typhoidal *Salmonella.*

The average economic burden per episode for iNTS disease is shown in figure 2a. As expected, the mean economic burden per episode was higher in UMICs than in LMICs and low-income countries. However, this appeared to be the opposite when comparing the aggregated iNTS disease economic burden with the total health expenditure by country. As shown in figure 2b, the proportion of the iNTS economic burden out of the total health expenditure was higher in sub-Saharan Africa than in other regions. In other words, while the iNTS disease economic burden per episode was lower in sub-Saharan Africa, its relative impact on the country’s total health expenditure would be greater in sub-Saharan Africa compared with other regions. Within the African region, the iNTS economic burden per episode was calculated to be higher in North Africa than in the rest of the African region, but the proportion of the economic burden out of the total health expenditure was higher in West and Central Africa than in North Africa.

**Figure 3:**
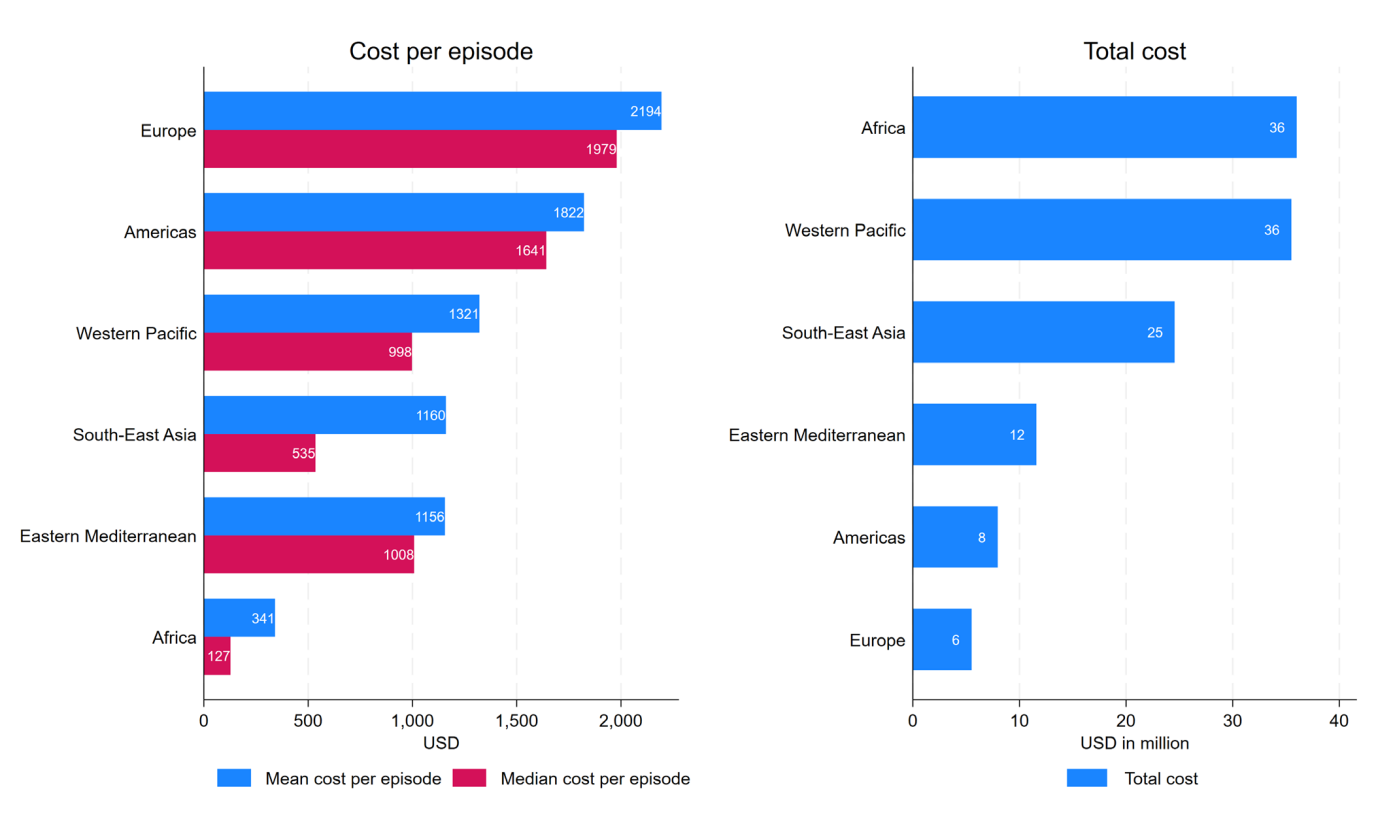
Economic burden of iNTS disease by WHO region (in US$). iNTS, invasive non-typhoidal *Salmonella.*

The average economic burden per episode and the total economic burden for iNTS disease are presented by WHO region in figure 3. The aggregated economic burden of iNTS disease was estimated by multiplying the number of iNTS disease cases per country, derived from the product of population and the incidence rate of iNTS disease,^22^ by the economic burden of iNTS disease per episode. While the mean economic burden per episode was the lowest in Africa and the highest in Europe, this was the opposite for the total economic burden due to iNTS disease, with Africa having the highest and Europe having the lowest burden. This is mainly because, despite the economic burden per episode being relatively low in Africa, the burden of iNTS disease was highly concentrated in the Africa region compared with the rest of the world, contributing to the overall high level of total economic burden of iNTS disease in Africa. At the country level, the highest aggregated economic burden was in China, estimated to be US$29.6 million, while the total economic burden was the lowest in Tuvalu, estimated to be US$27. The high economic burden of iNTS estimated in China was mainly driven by their large population size which resulted in the high number of iNTS cases despite the relatively low incidence rate.

## Discussion

The current study estimated the economic burden of iNTS disease at the global level. Given the nature of the right-skewed distribution of cost data, various regression models were considered to determine the most suitable model for the current cost data. The GLM gamma log model, which produced the most robust outcome, was selected based on a series of the model diagnostic tests and further used to predict the economic burden of iNTS disease and NTS disease for 123 countries. The highest economic burden per iNTS episode was found in Thailand, whereas Nigeria had the lowest iNTS economic burden per episode. While the economic burden per iNTS disease episode was generally lower in Africa than in other regions, its relative impact on society in terms of the total health expenditure by country is greater in Africa compared with the rest of the world. Within the sub-Saharan African region, the relative societal impact was the lowest in Comoros and Mauritius (less than the 5th percentiles) and the highest in Mali and Niger (greater than the 95th percentiles). The economic burden per iNTS disease episode was greater than the cost per NTS disease episode, as expected. Among the set of covariates included in the final model, LE was the most influential indicator in driving the estimated total cost apart from the binary iNTS variable. It is also worth noting that the aggregated economic burden of iNTS was sensitive to the country’s population size and incidence rate. For example, even if the incidence rate of iNTS disease was low in China, the total iNTS economic burden was estimated to be the highest due to the high number of iNTS disease cases driven by their large population size.

The economic burden of iNTS disease is substantial. For example, while the cost of malaria illness ranged from US$3.46 to US$81.08 in Mozambique^23^ and from US$15.12 per outpatient to US$27.21 per inpatient case in Uganda,^23^ the economic burden of iNTS disease was estimated to be US$92 and US$125 in Mozambique and Uganda, respectively. In addition, Baral *et al* reported the cost of illness for childhood diarrhoea to be US$36.56 per outpatient episode and US$159.90 per inpatient episode in LMICs.^24^ Although direct comparisons may not be applicable due to varying methodologies adopted, the current study estimated that the average economic burden per iNTS episode was US$341 in Africa and US$1160 in Southeast Asia.

Given the absence of primary economic burden data in many countries, it is not uncommon to use economic burden estimates from neighbouring countries or from the same region for broader economic evaluations such as a cost-effectiveness analysis at the regional level or global level. As previously indicated,^11^ the econometrically extrapolated cost estimates can be useful when there is a lack of quality (primary) data based on field-based prospective data collection or population-linked databases which are often missing and costly in the LMIC setting.

Some areas of uncertainty deserve attention. First, the current study used the cost data points from the existing studies identified in the recent systematic literature review. While the review was comprehensive, the number of data points may not be sufficient for the econometric models. In particular, the number of the primary data points on iNTS disease was lower compared with existing economic burden data on NTS disease. While field-based economic burden studies for iNTS disease were also included, the predicted model outcomes would be more robust with larger sample size (primary data points). Nonetheless, the current model passed a series of the model diagnostic tests including the distributional assumptions (ie, normality). Second, given that most of the cost data were not estimated at the country level but at the site, city or provincial level, one limitation is that the analysis was done assuming that these data could be generalised to the national level. A previous study indicated that while unit costs for medical services (DMC) may not be widely variable within a country, DNMC and IC may differ depending on household income levels.^25^ Generating more field-based economic burden data is critical to overcome the shortcomings of the current study. Third, it is also worth noting that the disease incidence of iNTS disease is closely associated with comorbidities such as HIV and malaria. Given that the current trajectories of the aggregated economic burden were estimated based on the assumption that existing healthcare programmes for controlling HIV or malaria were maintained, the withdrawal of existing international support or funding will likely affect the ongoing mitigation efforts on the comorbidities, which in turn, may cause the incidence and total economic burden of iNTS disease to increase. Fourth, the level of healthcare access and infrastructure, especially in the context of iNTS disease was unknown across countries; thus, the aggregated economic burden of iNTS disease might be overestimated. Fifth, country-level indicators such as GC do not capture variations within the subnational boundary level. This limits our understanding of the impact of iNTS disease economic burden on those with low socioeconomic status who may pay high out-of-pocket expenses.

Despite the limitations, this study rigorously compared the four econometric models and generated the estimates for 123 countries based on the model which met the statistical significance and the distribution of the current cost data. The model comparisons used in this study deliver an important message that a proper assessment on model specification is needed when dealing with cost data instead of adopting the most commonly used model assuming the same distributional assumption.^16^

There is no commercially available vaccine against iNTS disease. While there are several candidates in preclinical and early phase development,^6^ the societal and economic impacts of vaccination are largely uncertain given the lack of economic evaluation studies. Understanding the economic burden of iNTS disease is important to make a standardised comparison among other health problems competing for countries’ resources. In addition, the substantial economic burden of iNTS disease may be helpful to facilitate priority setting for vaccine investment. In countries where field-based economic burden studies for iNTS disease are not available, the cost extrapolation through the econometric framework can be helpful to understand what costs might be for the disease. Estimating the robust economic burden of iNTS disease is an essential step to assess the value of iNTS-containing vaccines and to measure the cost-effectiveness of various public health interventions, including future vaccination. It should be noted that primary data, being firsthand information, provides a more accurate and reliable representation of the real-world scenarios that the model aims to predict or understand. Thus, future research is needed to generate more field-based data which will make the model generalise better to unseen data and become less susceptible to noise or outliers.

## Supplementary material

10.1136/bmjgh-2025-019370 online supplemental file 1
