# Supplementary material for: Estimating the economic burden of invasive non-typhoidal Salmonella infections in low- and middle-income countries
Source: BMJ Glob Health. 2025 Nov 8;10(11):e019370. doi: 10.1136/bmjgh-2025-019370 (PMC12603720; doi:10.1136/bmjgh-2025-019370)
Supplement: online supplemental file 1 [file bmjgh-10-11-s001.docx]

**Supplementary information**

**Supplementary Figure 1. Residuals versus fitter values**


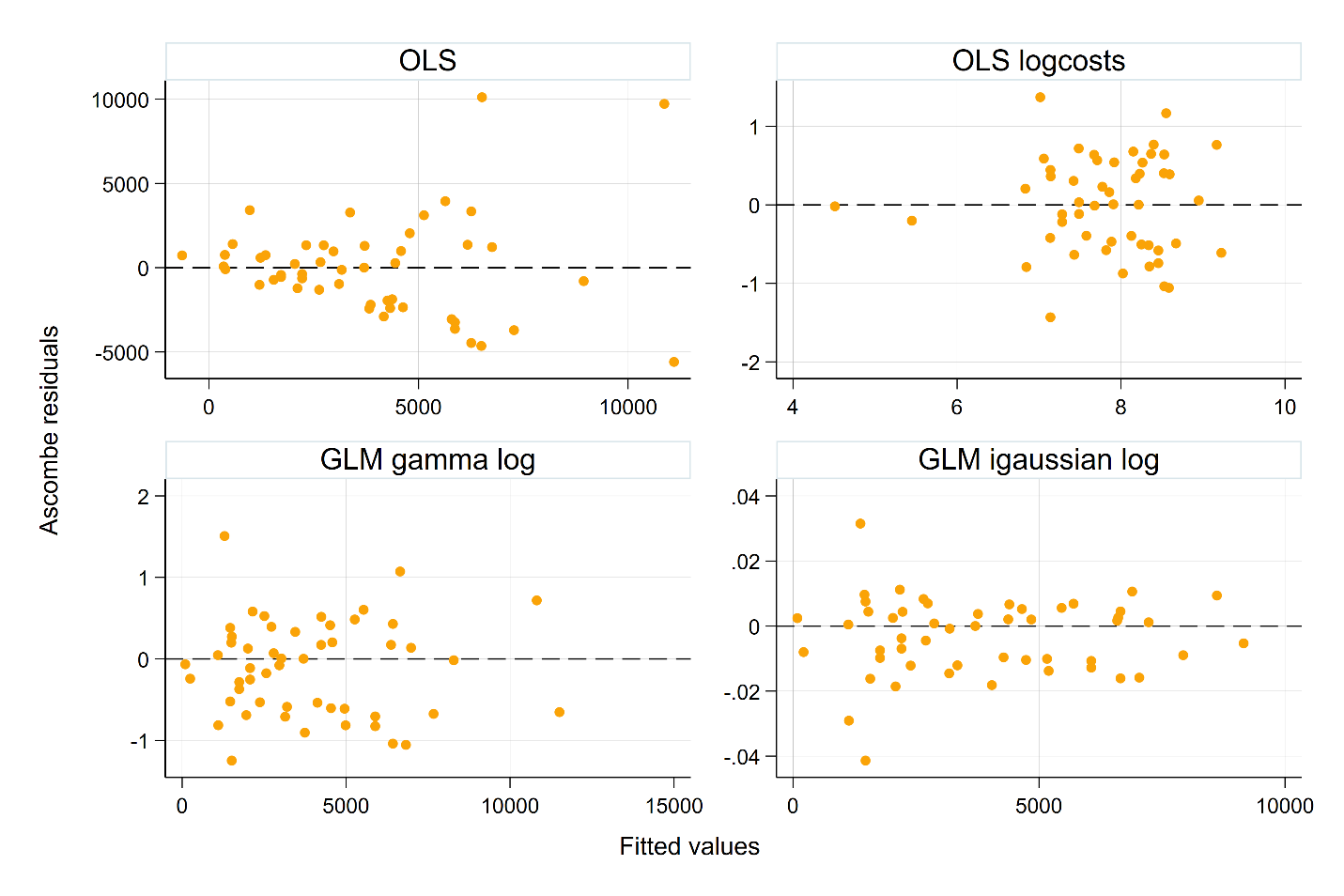


**Supplementary Table 1. Model performance**

| **Model type** | **MEAN** | **SD** | **MAE** | **RMSE** |
| --- | --- | --- | --- | --- |
| OLS | 3,881 | 2,564 | 2,064 | 3,159 |
| OLS: log, exp | 3,229 | 2,128 | 1,934 | 3,231 |
| OLS: log, smearing | 3,859 | 2,543 | 1,971 | 3,111 |
| GLM: gamma, log | 3,881 | 2,519 | 1,929 | 3,083 |
| GLM: inv.gaussian, log | 3,858 | 2,290 | 1,967 | 3,190 |

SD: standard deviation, MAE: mean absolute error, RMSE: root mean squared error

**Supplementary Table 2. Regression outputs (no adjustment)**

| **Variable** | **OLS** | | | **OLS with log costs** | | | **GLM (gamma, log)** | | | **GLM (inv.gaussian, log)** | | |
| --- | --- | --- | --- | --- | --- | --- | --- | --- | --- | --- | --- | --- |
|  | **Coefficients** | **SE** | **P-value** | **Coefficients** | **SE** | **P-value** | **Coefficients** | **SE** | **P-value** | **Coefficients** | **SE** | **P-value** |
| Constant | -31,326.838 | 13,046.5 | 0.021 | -9.458 | 2.7 | 0.001 | -9.237 | 2.7 | 0.001 | -8.609 | 1.8 | 0.000 |
| GDP capita | 1.735 | 0.7 | 0.012 | 0.001 | 0.0 | 0.000 | 0.001 | 0.0 | 0.000 | 0.001 | 0.0 | 0.001 |
| Life expectancy | 429.880 | 176.5 | 0.019 | 0.222 | 0.0 | 0.000 | 0.221 | 0.0 | 0.000 | 0.214 | 0.0 | 0.000 |
| Pop.density | -7.915 | 3.6 | 0.034 | -0.003 | 0.0 | 0.001 | -0.003 | 0.0 | 0.001 | -0.003 | 0.0 | 0.000 |
| iNTS | 5,225.817 | 1,525.9 | 0.001 | 0.768 | 0.3 | 0.019 | 0.670 | 0.3 | 0.028 | 0.413 | 0.3 | 0.181 |
| GDPcap&LE | -0.021 | 0.0 | 0.015 | 0.000 | 0.0 | 0.000 | 0.000 | 0.0 | 0.000 | 0.000 | 0.0 | 0.001 |
| Sq.density | 0.001 | 0.0 | 0.032 | 0.000 | 0.0 | 0.001 | 0.000 | 0.0 | 0.001 | 0.000 | 0.0 | 0.000 |
| AIC | 973.1 | | | Not comparable^a^ | | | 932.2 | | | 1,304.9 | | |
| SW test (p-value) | 0.001 | | | 0.596 | | | 0.408 | | | 0.025 | | |

^a^ The AIC generated by fitting the log transformed outcome variable is not comparable to the rest of the models as the response variable is different.

**Supplementary Table 3. Regression outputs (bootstrapping)**

| **Variable** | **OLS** | | | **OLS with log costs** | | | **GLM (gamma, log)** | | | **GLM (inv.gaussian, log)** | | |
| --- | --- | --- | --- | --- | --- | --- | --- | --- | --- | --- | --- | --- |
|  | **Coefficients** | **SE** | **P-value** | **Coefficients** | **SE** | **P-value** | **Coefficients** | **SE** | **P-value** | **Coefficients** | **SE** | **P-value** |
| Constant | -31,326.838 | 15,396.7 | 0.042 | -9.458 | 2.9 | 0.001 | -9.237 | 3.0 | 0.002 | -8.609 | 4.6 | 0.061 |
| GDP capita | 1.735 | 0.7 | 0.017 | 0.001 | 0.0 | 0.000 | 0.001 | 0.0 | 0.000 | 0.001 | 0.0 | 0.062 |
| Life expectancy | 429.880 | 206.1 | 0.037 | 0.222 | 0.0 | 0.000 | 0.221 | 0.0 | 0.000 | 0.214 | 0.1 | 0.001 |
| Pop.density | -7.915 | 4.5 | 0.081 | -0.003 | 0.0 | 0.098 | -0.003 | 0.0 | 0.079 | -0.003 | 0.0 | 0.304 |
| iNTS | 5,225.817 | 2,975.9 | 0.079 | 0.768 | 0.3 | 0.015 | 0.670 | 0.3 | 0.034 | 0.413 | 0.6 | 0.510 |
| GDPcap&LE | -0.021 | 0.0 | 0.022 | 0.000 | 0.0 | 0.000 | 0.000 | 0.0 | 0.000 | 0.000 | 0.0 | 0.059 |
| Sq.density | 0.001 | 0.0 | 0.900 | 0.000 | 0.0 | 0.908 | 0.000 | 0.0 | 0.898 | 0.000 | 0.0 | 0.921 |
| AIC | 973.1 | | | Not comparable^a^ | | | 932.2 | | | 1,304.9 | | |
| SW test (p-value) | 0.001 | | | 0.596 | | | 0.408 | | | 0.025 | | |

^a^ The AIC generated by fitting the log transformed outcome variable is not comparable to the rest of the models as the response variable is different.

**Supplementary Table 4. Regression outputs with no imputed costs (no adjustment)**

| **Variable** | **OLS** | | | **OLS with log costs** | | | **GLM (gamma, log)** | | | **GLM (inv.gaussian, log)** | | |
| --- | --- | --- | --- | --- | --- | --- | --- | --- | --- | --- | --- | --- |
|  | **Coefficients** | **SE** | **P-value** | **Coefficients** | **SE** | **P-value** | **Coefficients** | **SE** | **P-value** | **Coefficients** | **SE** | **P-value** |
| Constant | -23,223.830 | 12,391.5 | 0.068 | -7.419 | 3.5 | 0.042 | -6.846 | 3.4 | 0.042 | -6.756 | 1.9 | 0.000 |
| GDP capita | 1.871 | 0.6 | 0.005 | 0.001 | 0.0 | 0.001 | 0.001 | 0.0 | 0.000 | 0.001 | 0.0 | 0.001 |
| Life expectancy | 308.015 | 167.6 | 0.073 | 0.190 | 0.0 | 0.000 | 0.184 | 0.0 | 0.000 | 0.184 | 0.0 | 0.000 |
| Pop.density | -5.714 | 3.4 | 0.104 | -0.003 | 0.0 | 0.007 | -0.002 | 0.0 | 0.007 | -0.003 | 0.0 | 0.001 |
| iNTS | 4,724.833 | 1,449.3 | 0.002 | 0.863 | 0.4 | 0.043 | 0.656 | 0.4 | 0.062 | 0.329 | 0.3 | 0.320 |
| GDPcap&LE | -0.023 | 0.0 | 0.006 | 0.000 | 0.0 | 0.001 | 0.000 | 0.0 | 0.000 | 0.000 | 0.0 | 0.001 |
| Sq.density | 0.001 | 0.0 | 0.100 | 0.000 | 0.0 | 0.006 | 0.000 | 0.0 | 0.007 | 0.000 | 0.0 | 0.001 |
| AIC | 967.9 | | | Not comparable^a^ | | | 908.7 | | | 1,256.1 | | |
| SW test (p-value) | 0.000 | | | 0.051 | | | 0.985 | | | 0.005 | | |

^a^ The AIC generated by fitting the log transformed outcome variable is not comparable to the rest of the models as the response variable is different.

**Supplementary Table 5. Regression outputs with no imputed costs (Huber/White estimator)**

| **Variable** | **OLS** | | | **OLS with log costs** | | | **GLM (gamma, log)** | | | **GLM (inv.gaussian, log)** | | |
| --- | --- | --- | --- | --- | --- | --- | --- | --- | --- | --- | --- | --- |
|  | **Coefficients** | **SE** | **P-value** | **Coefficients** | **SE** | **P-value** | **Coefficients** | **SE** | **P-value** | **Coefficients** | **SE** | **P-value** |
| Constant | -23,223.830 | 11,905.4 | 0.057 | -7.419 | 1.9 | 0.000 | -6.846 | 2.0 | 0.001 | -6.756 | 1.0 | 0.000 |
| GDP capita | 1.871 | 0.7 | 0.013 | 0.001 | 0.0 | 0.004 | 0.001 | 0.0 | 0.000 | 0.001 | 0.0 | 0.001 |
| Life expectancy | 308.015 | 157.9 | 0.058 | 0.190 | 0.0 | 0.000 | 0.184 | 0.0 | 0.000 | 0.184 | 0.0 | 0.000 |
| Pop.density | -5.714 | 2.1 | 0.011 | -0.003 | 0.0 | 0.009 | -0.002 | 0.0 | 0.001 | -0.003 | 0.0 | 0.004 |
| iNTS | 4,724.833 | 2,661.4 | 0.083 | 0.863 | 0.3 | 0.004 | 0.656 | 0.3 | 0.013 | 0.329 | 0.2 | 0.170 |
| GDPcap&LE | -0.023 | 0.0 | 0.015 | 0.000 | 0.0 | 0.004 | 0.000 | 0.0 | 0.000 | 0.000 | 0.0 | 0.001 |
| Sq.density | 0.001 | 0.0 | 0.010 | 0.000 | 0.0 | 0.008 | 0.000 | 0.0 | 0.001 | 0.000 | 0.0 | 0.005 |
| AIC | 967.9 | | | Not comparable^a^ | | | 906.7 | | | 1,254.1 | | |
| SW test (p-value) | 0.000 | | | 0.051 | | | 0.985 | | | 0.005 | | |

^a^ The AIC generated by fitting the log transformed outcome variable is not comparable to the rest of the models as the response variable is different.

**Supplementary Table 6. Regression outputs with no imputed costs (bootstrapping)**

| **Variable** | **OLS** | | | **OLS with log costs** | | | **GLM (gamma, log)** | | | **GLM (inv.gaussian, log)** | | |
| --- | --- | --- | --- | --- | --- | --- | --- | --- | --- | --- | --- | --- |
|  | **Coefficients** | **SE** | **P-value** | **Coefficients** | **SE** | **P-value** | **Coefficients** | **SE** | **P-value** | **Coefficients** | **SE** | **P-value** |
| Constant | -23,223.830 | 15,305.0 | 0.129 | -7.419 | 4.1 | 0.073 | -6.846 | 4.6 | 0.137 | -6.756 | 7.6 | 0.376 |
| GDP capita | 1.871 | 0.7 | 0.008 | 0.001 | 0.0 | 0.008 | 0.001 | 0.0 | 0.002 | 0.001 | 0.0 | 0.158 |
| Life expectancy | 308.015 | 204.5 | 0.132 | 0.190 | 0.1 | 0.001 | 0.184 | 0.1 | 0.003 | 0.184 | 0.1 | 0.072 |
| Pop.density | -5.714 | 4.0 | 0.158 | -0.003 | 0.0 | 0.153 | -0.002 | 0.0 | 0.189 | -0.003 | 0.0 | 0.325 |
| iNTS | 4,724.833 | 3,056.4 | 0.122 | 0.863 | 0.4 | 0.016 | 0.656 | 0.4 | 0.080 | 0.329 | 0.8 | 0.664 |
| GDPcap&LE | -0.023 | 0.0 | 0.011 | 0.000 | 0.0 | 0.010 | 0.000 | 0.0 | 0.002 | 0.000 | 0.0 | 0.164 |
| Sq.density | 0.001 | 0.0 | 0.923 | 0.000 | 0.0 | 0.909 | 0.000 | 0.0 | 0.933 | 0.000 | 0.0 | 0.946 |
| AIC | 967.9 | | | 135.7 | | | 908.7 | | | 1,256.1 | | |
| SW test (p-value) | 0.000 | | | 0.051 | | | 0.985 | | | 0.005 | | |

^a^ The AIC generated by fitting the log transformed outcome variable is not comparable to the rest of the models as the response variable is different.

**Supplementary Table 7. Estimated economic burden per iNTS episode by country**

| **Country** | **Region** | **Value** | **95% confidence interval** | | **Value** | **95% confidence interval** | |
| --- | --- | --- | --- | --- | --- | --- | --- |
|  |  | **US$** | **Lower** | **Upper** | **I$** | **Lower** | **Upper** |
| Afghanistan | South Asia | 152 | 78 | 227 | 25 | 13 | 38 |
| Albania | Europe & Central Asia | 3,875 | 1,525 | 6,226 | 1,520 | 598 | 2,441 |
| Algeria | Middle East & North Africa | 4,376 | 1,731 | 7,020 | 1,256 | 497 | 2,015 |
| Angola | Sub-Saharan Africa | 191 | 97 | 285 | 50 | 25 | 74 |
| Argentina | Latin America & Caribbean | 4,716 | 1,981 | 7,450 | 1,925 | 809 | 3,042 |
| Armenia | Europe & Central Asia | 1,659 | 812 | 2,506 | 488 | 239 | 737 |
| Azerbaijan | Europe & Central Asia | 984 | 518 | 1,450 | 265 | 139 | 390 |
| Bangladesh | South Asia | 122 | 59 | 185 | 41 | 20 | 62 |
| Belarus | Europe & Central Asia | 2,345 | 1,135 | 3,556 | 636 | 308 | 965 |
| Belize | Latin America & Caribbean | 1,677 | 861 | 2,493 | 879 | 451 | 1,307 |
| Benin | Sub-Saharan Africa | 96 | 46 | 146 | 37 | 17 | 56 |
| Bhutan | South Asia | 1,746 | 862 | 2,631 | 463 | 229 | 698 |
| Bolivia | Latin America & Caribbean | 359 | 190 | 528 | 129 | 68 | 189 |
| Bosnia and Herzegovina | Europe & Central Asia | 3,611 | 1,525 | 5,697 | 1,420 | 600 | 2,241 |
| Botswana | Sub-Saharan Africa | 388 | 193 | 582 | 169 | 84 | 253 |
| Brazil | Latin America & Caribbean | 2,665 | 1,271 | 4,060 | 1,176 | 561 | 1,791 |
| Burkina Faso | Sub-Saharan Africa | 87 | 40 | 133 | 31 | 15 | 48 |
| Burundi | Sub-Saharan Africa | 49 | 25 | 74 | 13 | 6 | 19 |
| Cabo Verde | Sub-Saharan Africa | 1,992 | 898 | 3,087 | 1,029 | 464 | 1,595 |
| Cambodia | East Asia & Pacific | 814 | 427 | 1,202 | 284 | 149 | 419 |
| Cameroon | Sub-Saharan Africa | 130 | 63 | 197 | 45 | 22 | 69 |
| Central African Republic | Sub-Saharan Africa | 30 | 10 | 51 | 13 | 4 | 22 |
| Chad | Sub-Saharan Africa | 23 | 7 | 40 | 9 | 3 | 15 |
| China | East Asia & Pacific | 5,187 | 1,798 | 8,576 | 3,207 | 1,112 | 5,302 |
| Colombia | Latin America & Caribbean | 2,349 | 1,117 | 3,581 | 826 | 393 | 1,259 |
| Comoros | Sub-Saharan Africa | 96 | 50 | 141 | 44 | 23 | 65 |
| Congo, Dem. Rep. | Sub-Saharan Africa | 90 | 42 | 138 | 38 | 18 | 59 |
| Congo, Rep. | Sub-Saharan Africa | 304 | 160 | 447 | 122 | 64 | 179 |
| Costa Rica | Latin America & Caribbean | 5,034 | 1,908 | 8,161 | 2,677 | 1,015 | 4,340 |
| Cote d'Ivoire | Sub-Saharan Africa | 98 | 44 | 151 | 40 | 18 | 61 |
| Djibouti | Middle East & North Africa | 242 | 125 | 359 | 123 | 63 | 182 |
| Dominica | Latin America & Caribbean | 2,237 | 1,064 | 3,410 | 1,185 | 564 | 1,807 |
| Dominican Republic | Latin America & Caribbean | 1,606 | 770 | 2,442 | 621 | 298 | 944 |
| Ecuador | Latin America & Caribbean | 2,523 | 1,157 | 3,889 | 1,132 | 519 | 1,745 |
| Egypt, Arab Rep. | Middle East & North Africa | 1,059 | 547 | 1,572 | 260 | 134 | 386 |
| El Salvador | Latin America & Caribbean | 764 | 389 | 1,139 | 328 | 167 | 489 |
| Equatorial Guinea | Sub-Saharan Africa | 320 | 156 | 483 | 132 | 65 | 199 |
| Eswatini | Sub-Saharan Africa | 95 | 39 | 150 | 38 | 16 | 60 |
| Ethiopia | Sub-Saharan Africa | 278 | 149 | 406 | 98 | 53 | 143 |
| Fiji | East Asia & Pacific | 730 | 395 | 1,066 | 321 | 173 | 468 |
| Gabon | Sub-Saharan Africa | 980 | 530 | 1,430 | 433 | 234 | 631 |
| Gambia, The | Sub-Saharan Africa | 99 | 51 | 148 | 28 | 15 | 42 |
| Georgia | Europe & Central Asia | 1,700 | 842 | 2,558 | 457 | 226 | 688 |
| Ghana | Sub-Saharan Africa | 235 | 125 | 345 | 87 | 46 | 128 |
| Grenada | Latin America & Caribbean | 1,727 | 744 | 2,710 | 1,086 | 468 | 1,704 |
| Guatemala | Latin America & Caribbean | 848 | 447 | 1,249 | 352 | 186 | 519 |
| Guinea | Sub-Saharan Africa | 90 | 41 | 138 | 29 | 13 | 44 |
| Guinea-Bissau | Sub-Saharan Africa | 95 | 45 | 145 | 35 | 16 | 53 |
| Haiti | Latin America & Caribbean | 100 | 52 | 147 | 58 | 31 | 86 |
| Honduras | Latin America & Caribbean | 1,000 | 518 | 1,482 | 441 | 228 | 654 |
| India | South Asia | 211 | 114 | 308 | 59 | 32 | 86 |
| Indonesia | East Asia & Pacific | 606 | 327 | 886 | 204 | 110 | 298 |
| Iran, Islamic Rep. | Middle East & North Africa | 2,498 | 1,135 | 3,861 | 678 | 308 | 1,048 |
| Iraq | Middle East & North Africa | 1,195 | 615 | 1,774 | 457 | 235 | 679 |
| Jamaica | Latin America & Caribbean | 842 | 432 | 1,252 | 454 | 233 | 675 |
| Jordan | Middle East & North Africa | 2,232 | 996 | 3,469 | 1,017 | 454 | 1,580 |
| Kazakhstan | Europe & Central Asia | 2,257 | 1,166 | 3,349 | 684 | 353 | 1,015 |
| Kenya | Sub-Saharan Africa | 157 | 79 | 236 | 61 | 31 | 91 |
| Kiribati | East Asia & Pacific | 444 | 240 | 649 | 354 | 191 | 517 |
| Kyrgyz Republic | Europe & Central Asia | 1,511 | 743 | 2,278 | 356 | 175 | 536 |
| Lao PDR | East Asia & Pacific | 755 | 405 | 1,105 | 236 | 127 | 346 |
| Lebanon | Middle East & North Africa | 956 | 410 | 1,503 | 334 | 143 | 524 |
| Lesotho | Sub-Saharan Africa | 24 | 7 | 41 | 10 | 3 | 17 |
| Liberia | Sub-Saharan Africa | 124 | 61 | 187 | 54 | 26 | 81 |
| Libya | Middle East & North Africa | 2,196 | 1,080 | 3,312 | 857 | 422 | 1,293 |
| Madagascar | Sub-Saharan Africa | 276 | 148 | 403 | 84 | 45 | 124 |
| Malawi | Sub-Saharan Africa | 132 | 69 | 195 | 48 | 25 | 71 |
| Malaysia | East Asia & Pacific | 3,581 | 1,547 | 5,615 | 1,309 | 566 | 2,053 |
| Maldives | South Asia | 310 | 92 | 529 | 166 | 49 | 282 |
| Mali | Sub-Saharan Africa | 95 | 44 | 147 | 35 | 16 | 54 |
| Marshall Islands | East Asia & Pacific | 385 | 208 | 562 | 358 | 193 | 523 |
| Mauritania | Sub-Saharan Africa | 368 | 197 | 538 | 123 | 66 | 180 |
| Mauritius | Sub-Saharan Africa | 779 | 357 | 1,201 | 311 | 142 | 479 |
| Mexico | Latin America & Caribbean | 1,887 | 975 | 2,799 | 925 | 478 | 1,373 |
| Micronesia, Fed. Sts. | East Asia & Pacific | 998 | 509 | 1,487 | 977 | 499 | 1,456 |
| Moldova | Europe & Central Asia | 957 | 508 | 1,406 | 322 | 171 | 473 |
| Mongolia | East Asia & Pacific | 1,700 | 861 | 2,538 | 519 | 263 | 775 |
| Montenegro | Europe & Central Asia | 3,292 | 1,499 | 5,084 | 1,271 | 579 | 1,963 |
| Morocco | Middle East & North Africa | 2,361 | 1,064 | 3,657 | 1,036 | 467 | 1,606 |
| Mozambique | Sub-Saharan Africa | 92 | 43 | 141 | 32 | 15 | 49 |
| Myanmar | East Asia & Pacific | 354 | 191 | 516 | 85 | 46 | 124 |
| Namibia | Sub-Saharan Africa | 198 | 92 | 305 | 90 | 42 | 138 |
| Nepal | South Asia | 463 | 247 | 679 | 128 | 68 | 187 |
| Nicaragua | Latin America & Caribbean | 2,230 | 1,015 | 3,446 | 670 | 305 | 1,035 |
| Niger | Sub-Saharan Africa | 160 | 81 | 239 | 61 | 31 | 92 |
| Nigeria | Sub-Saharan Africa | 18 | 5 | 31 | 7 | 2 | 11 |
| North Macedonia | Europe & Central Asia | 2,960 | 1,302 | 4,618 | 1,008 | 443 | 1,573 |
| Pakistan | South Asia | 232 | 126 | 338 | 63 | 34 | 92 |
| Palestine | Middle East & North Africa | 391 | 181 | 601 | 254 | 117 | 390 |
| Papua New Guinea | East Asia & Pacific | 453 | 245 | 662 | 296 | 160 | 433 |
| Paraguay | Latin America & Caribbean | 1,587 | 819 | 2,355 | 616 | 318 | 914 |
| Peru | Latin America & Caribbean | 2,348 | 1,136 | 3,560 | 1,049 | 507 | 1,591 |
| Philippines | East Asia & Pacific | 437 | 230 | 643 | 172 | 91 | 253 |
| Rwanda | Sub-Saharan Africa | 122 | 66 | 177 | 37 | 20 | 54 |
| Samoa | East Asia & Pacific | 1,872 | 892 | 2,852 | 1,182 | 564 | 1,801 |
| Sao Tome and Principe | Sub-Saharan Africa | 406 | 219 | 593 | 167 | 90 | 245 |
| Senegal | Sub-Saharan Africa | 492 | 266 | 718 | 188 | 102 | 275 |
| Serbia | Europe & Central Asia | 2,509 | 1,197 | 3,820 | 1,026 | 490 | 1,562 |
| Sierra Leone | Sub-Saharan Africa | 89 | 43 | 135 | 28 | 13 | 42 |
| Solomon Islands | East Asia & Pacific | 1,195 | 615 | 1,775 | 1,009 | 519 | 1,498 |
| Somalia | Sub-Saharan Africa | 39 | 14 | 64 | 15 | 6 | 25 |
| South Africa | Sub-Saharan Africa | 413 | 213 | 612 | 206 | 106 | 306 |
| Sri Lanka | South Asia | 1,899 | 750 | 3,047 | 530 | 210 | 851 |
| St. Lucia | Latin America & Caribbean | 1,162 | 586 | 1,738 | 636 | 321 | 952 |
| St. Vincent and the Grenadines | Latin America & Caribbean | 910 | 476 | 1,343 | 485 | 254 | 717 |
| Sudan | Sub-Saharan Africa | 359 | 193 | 524 | 79 | 42 | 115 |
| Suriname | Latin America & Caribbean | 1,517 | 783 | 2,251 | 413 | 213 | 613 |
| Tajikistan | Europe & Central Asia | 1,259 | 626 | 1,892 | 283 | 141 | 426 |
| Tanzania | Sub-Saharan Africa | 404 | 219 | 589 | 134 | 73 | 196 |
| Thailand | East Asia & Pacific | 5,269 | 1,752 | 8,786 | 1,837 | 611 | 3,063 |
| Timor-Leste | East Asia & Pacific | 622 | 335 | 910 | 245 | 132 | 358 |
| Togo | Sub-Saharan Africa | 119 | 60 | 178 | 43 | 22 | 65 |
| Tonga | East Asia & Pacific | 1,159 | 587 | 1,731 | 757 | 383 | 1,130 |
| Tunisia | Middle East & North Africa | 2,266 | 1,034 | 3,497 | 711 | 325 | 1,098 |
| Turkiye | Europe & Central Asia | 3,930 | 1,589 | 6,271 | 1,210 | 489 | 1,931 |
| Tuvalu | East Asia & Pacific | 221 | 118 | 323 | 233 | 125 | 342 |
| Uganda | Sub-Saharan Africa | 125 | 65 | 185 | 41 | 21 | 61 |
| Ukraine | Europe & Central Asia | 1,116 | 584 | 1,648 | 299 | 156 | 441 |
| Uzbekistan | Europe & Central Asia | 1,134 | 576 | 1,692 | 277 | 141 | 413 |
| Vanuatu | East Asia & Pacific | 1,292 | 664 | 1,920 | 1,273 | 654 | 1,892 |
| Vietnam | East Asia & Pacific | 1,230 | 566 | 1,895 | 378 | 174 | 583 |
| Zambia | Sub-Saharan Africa | 158 | 79 | 236 | 51 | 25 | 76 |
| Zimbabwe | Sub-Saharan Africa | 110 | 51 | 169 | 60 | 28 | 91 |

**Supplementary** **Table 8. Estimated economic burden per NTS episode by country**

| **Country** | **Region** | **Value** | **95% confidence interval** | | **Value** | **95% confidence interval** | |
| --- | --- | --- | --- | --- | --- | --- | --- |
|  |  | **US$** | **Lower** | **Upper** | **I$** | **Lower** | **Upper** |
| Afghanistan | South Asia | 78 | 23 | 132 | 13 | 4 | 22 |
| Albania | Europe & Central Asia | 1,983 | 957 | 3,009 | 778 | 375 | 1,180 |
| Algeria | Middle East & North Africa | 2,239 | 1,082 | 3,396 | 643 | 310 | 975 |
| Angola | Sub-Saharan Africa | 98 | 28 | 167 | 25 | 7 | 43 |
| Argentina | Latin America & Caribbean | 2,413 | 1,177 | 3,648 | 985 | 481 | 1,490 |
| Armenia | Europe & Central Asia | 849 | 410 | 1,288 | 250 | 121 | 379 |
| Azerbaijan | Europe & Central Asia | 503 | 229 | 778 | 135 | 62 | 209 |
| Bangladesh | South Asia | 62 | 30 | 95 | 21 | 10 | 32 |
| Belarus | Europe & Central Asia | 1,200 | 581 | 1,819 | 326 | 158 | 493 |
| Belize | Latin America & Caribbean | 858 | 402 | 1,314 | 450 | 211 | 689 |
| Benin | Sub-Saharan Africa | 49 | 12 | 87 | 19 | 4 | 33 |
| Bhutan | South Asia | 894 | 430 | 1,357 | 237 | 114 | 360 |
| Bolivia | Latin America & Caribbean | 184 | 63 | 305 | 66 | 22 | 109 |
| Bosnia and Herzegovina | Europe & Central Asia | 1,848 | 902 | 2,793 | 727 | 355 | 1,099 |
| Botswana | Sub-Saharan Africa | 198 | 55 | 342 | 86 | 24 | 149 |
| Brazil | Latin America & Caribbean | 1,364 | 663 | 2,064 | 602 | 293 | 910 |
| Burkina Faso | Sub-Saharan Africa | 44 | 10 | 79 | 16 | 4 | 28 |
| Burundi | Sub-Saharan Africa | 25 | 7 | 43 | 6 | 2 | 11 |
| Cabo Verde | Sub-Saharan Africa | 1,019 | 499 | 1,539 | 527 | 258 | 795 |
| Cambodia | East Asia & Pacific | 417 | 191 | 643 | 145 | 66 | 224 |
| Cameroon | Sub-Saharan Africa | 66 | 17 | 116 | 23 | 6 | 40 |
| Central African Republic | Sub-Saharan Africa | 16 | 1 | 30 | 7 | 0 | 13 |
| Chad | Sub-Saharan Africa | 12 | 0 | 24 | 5 | 0 | 9 |
| China | East Asia & Pacific | 2,654 | 1,240 | 4,068 | 1,641 | 766 | 2,516 |
| Colombia | Latin America & Caribbean | 1,202 | 585 | 1,819 | 423 | 206 | 639 |
| Comoros | Sub-Saharan Africa | 49 | 16 | 81 | 23 | 8 | 37 |
| Congo, Dem. Rep. | Sub-Saharan Africa | 46 | 10 | 82 | 20 | 4 | 35 |
| Congo, Rep. | Sub-Saharan Africa | 155 | 53 | 258 | 62 | 21 | 104 |
| Costa Rica | Latin America & Caribbean | 2,576 | 1,233 | 3,919 | 1,370 | 656 | 2,084 |
| Cote d'Ivoire | Sub-Saharan Africa | 50 | 10 | 90 | 20 | 4 | 36 |
| Djibouti | Middle East & North Africa | 124 | 38 | 209 | 63 | 19 | 106 |
| Dominica | Latin America & Caribbean | 1,145 | 557 | 1,732 | 606 | 295 | 918 |
| Dominican Republic | Latin America & Caribbean | 822 | 399 | 1,244 | 318 | 154 | 481 |
| Ecuador | Latin America & Caribbean | 1,291 | 632 | 1,950 | 579 | 283 | 875 |
| Egypt, Arab Rep. | Middle East & North Africa | 542 | 253 | 832 | 133 | 62 | 204 |
| El Salvador | Latin America & Caribbean | 391 | 184 | 598 | 168 | 79 | 257 |
| Equatorial Guinea | Sub-Saharan Africa | 163 | 43 | 284 | 68 | 18 | 117 |
| Eswatini | Sub-Saharan Africa | 48 | 8 | 89 | 19 | 3 | 36 |
| Ethiopia | Sub-Saharan Africa | 142 | 53 | 231 | 50 | 19 | 81 |
| Fiji | East Asia & Pacific | 374 | 156 | 591 | 164 | 69 | 260 |
| Gabon | Sub-Saharan Africa | 501 | 197 | 806 | 221 | 87 | 356 |
| Gambia, The | Sub-Saharan Africa | 51 | 15 | 86 | 15 | 4 | 25 |
| Georgia | Europe & Central Asia | 870 | 418 | 1,322 | 234 | 112 | 355 |
| Ghana | Sub-Saharan Africa | 120 | 42 | 199 | 44 | 15 | 74 |
| Grenada | Latin America & Caribbean | 884 | 432 | 1,335 | 556 | 272 | 840 |
| Guatemala | Latin America & Caribbean | 434 | 197 | 671 | 180 | 82 | 279 |
| Guinea | Sub-Saharan Africa | 46 | 10 | 82 | 15 | 3 | 26 |
| Guinea-Bissau | Sub-Saharan Africa | 48 | 11 | 86 | 18 | 4 | 31 |
| Haiti | Latin America & Caribbean | 51 | 17 | 85 | 30 | 10 | 50 |
| Honduras | Latin America & Caribbean | 512 | 238 | 786 | 226 | 105 | 346 |
| India | South Asia | 108 | 45 | 171 | 30 | 13 | 47 |
| Indonesia | East Asia & Pacific | 310 | 132 | 488 | 104 | 44 | 164 |
| Iran, Islamic Rep. | Middle East & North Africa | 1,278 | 626 | 1,931 | 347 | 170 | 524 |
| Iraq | Middle East & North Africa | 611 | 286 | 937 | 234 | 109 | 358 |
| Jamaica | Latin America & Caribbean | 431 | 202 | 660 | 232 | 109 | 356 |
| Jordan | Middle East & North Africa | 1,142 | 560 | 1,725 | 520 | 255 | 786 |
| Kazakhstan | Europe & Central Asia | 1,155 | 538 | 1,772 | 350 | 163 | 537 |
| Kenya | Sub-Saharan Africa | 81 | 23 | 138 | 31 | 9 | 53 |
| Kiribati | East Asia & Pacific | 227 | 96 | 358 | 181 | 77 | 286 |
| Kyrgyz Republic | Europe & Central Asia | 773 | 372 | 1,174 | 182 | 88 | 276 |
| Lao PDR | East Asia & Pacific | 386 | 168 | 605 | 121 | 53 | 189 |
| Lebanon | Middle East & North Africa | 489 | 239 | 739 | 171 | 83 | 258 |
| Lesotho | Sub-Saharan Africa | 12 | 0 | 24 | 5 | 0 | 10 |
| Liberia | Sub-Saharan Africa | 63 | 17 | 110 | 28 | 7 | 48 |
| Libya | Middle East & North Africa | 1,124 | 541 | 1,706 | 439 | 211 | 666 |
| Madagascar | Sub-Saharan Africa | 141 | 51 | 231 | 43 | 16 | 71 |
| Malawi | Sub-Saharan Africa | 67 | 22 | 113 | 25 | 8 | 41 |
| Malaysia | East Asia & Pacific | 1,832 | 897 | 2,768 | 670 | 328 | 1,012 |
| Maldives | South Asia | 159 | 71 | 247 | 85 | 38 | 132 |
| Mali | Sub-Saharan Africa | 49 | 10 | 87 | 18 | 4 | 32 |
| Marshall Islands | East Asia & Pacific | 197 | 75 | 319 | 183 | 70 | 297 |
| Mauritania | Sub-Saharan Africa | 188 | 68 | 309 | 63 | 23 | 103 |
| Mauritius | Sub-Saharan Africa | 399 | 195 | 602 | 159 | 78 | 240 |
| Mexico | Latin America & Caribbean | 965 | 450 | 1,481 | 473 | 221 | 726 |
| Micronesia, Fed. Sts. | East Asia & Pacific | 511 | 241 | 781 | 500 | 236 | 764 |
| Moldova | Europe & Central Asia | 490 | 219 | 760 | 165 | 74 | 256 |
| Mongolia | East Asia & Pacific | 870 | 412 | 1,327 | 266 | 126 | 405 |
| Montenegro | Europe & Central Asia | 1,684 | 824 | 2,544 | 650 | 318 | 982 |
| Morocco | Middle East & North Africa | 1,208 | 592 | 1,824 | 530 | 260 | 801 |
| Mozambique | Sub-Saharan Africa | 47 | 11 | 83 | 16 | 4 | 29 |
| Myanmar | East Asia & Pacific | 181 | 70 | 292 | 43 | 17 | 70 |
| Namibia | Sub-Saharan Africa | 102 | 23 | 180 | 46 | 10 | 82 |
| Nepal | South Asia | 237 | 105 | 369 | 65 | 29 | 102 |
| Nicaragua | Latin America & Caribbean | 1,141 | 559 | 1,724 | 343 | 168 | 518 |
| Niger | Sub-Saharan Africa | 82 | 24 | 140 | 31 | 9 | 54 |
| Nigeria | Sub-Saharan Africa | 9 | 0 | 18 | 3 | 0 | 7 |
| North Macedonia | Europe & Central Asia | 1,515 | 742 | 2,287 | 516 | 253 | 779 |
| Pakistan | South Asia | 119 | 47 | 190 | 32 | 13 | 52 |
| Palestine | Middle East & North Africa | 200 | 98 | 302 | 130 | 63 | 196 |
| Papua New Guinea | East Asia & Pacific | 232 | 89 | 375 | 152 | 58 | 245 |
| Paraguay | Latin America & Caribbean | 812 | 379 | 1,246 | 315 | 147 | 483 |
| Peru | Latin America & Caribbean | 1,201 | 582 | 1,821 | 537 | 260 | 813 |
| Philippines | East Asia & Pacific | 223 | 101 | 345 | 88 | 40 | 136 |
| Rwanda | Sub-Saharan Africa | 62 | 25 | 100 | 19 | 8 | 30 |
| Samoa | East Asia & Pacific | 958 | 466 | 1,450 | 605 | 294 | 916 |
| Sao Tome and Principe | Sub-Saharan Africa | 208 | 89 | 327 | 86 | 37 | 135 |
| Senegal | Sub-Saharan Africa | 252 | 105 | 398 | 96 | 40 | 152 |
| Serbia | Europe & Central Asia | 1,284 | 624 | 1,943 | 525 | 255 | 795 |
| Sierra Leone | Sub-Saharan Africa | 45 | 11 | 80 | 14 | 3 | 25 |
| Solomon Islands | East Asia & Pacific | 611 | 286 | 937 | 516 | 241 | 791 |
| Somalia | Sub-Saharan Africa | 20 | 2 | 38 | 8 | 1 | 15 |
| South Africa | Sub-Saharan Africa | 211 | 65 | 357 | 105 | 33 | 178 |
| Sri Lanka | South Asia | 972 | 469 | 1,474 | 271 | 131 | 412 |
| St. Lucia | Latin America & Caribbean | 595 | 283 | 907 | 326 | 155 | 497 |
| St. Vincent and the Grenadines | Latin America & Caribbean | 466 | 213 | 718 | 248 | 114 | 383 |
| Sudan | Sub-Saharan Africa | 183 | 70 | 297 | 40 | 15 | 65 |
| Suriname | Latin America & Caribbean | 776 | 362 | 1,190 | 212 | 99 | 324 |
| Tajikistan | Europe & Central Asia | 644 | 309 | 980 | 145 | 69 | 220 |
| Tanzania | Sub-Saharan Africa | 207 | 83 | 331 | 69 | 27 | 110 |
| Thailand | East Asia & Pacific | 2,696 | 1,243 | 4,149 | 940 | 433 | 1,447 |
| Timor-Leste | East Asia & Pacific | 318 | 137 | 500 | 125 | 54 | 197 |
| Togo | Sub-Saharan Africa | 61 | 18 | 104 | 22 | 6 | 38 |
| Tonga | East Asia & Pacific | 593 | 281 | 905 | 387 | 184 | 591 |
| Tunisia | Middle East & North Africa | 1,159 | 567 | 1,751 | 364 | 178 | 550 |
| Turkiye | Europe & Central Asia | 2,011 | 976 | 3,046 | 619 | 300 | 938 |
| Tuvalu | East Asia & Pacific | 113 | 41 | 185 | 119 | 43 | 195 |
| Uganda | Sub-Saharan Africa | 64 | 20 | 107 | 21 | 7 | 35 |
| Ukraine | Europe & Central Asia | 571 | 262 | 880 | 153 | 70 | 236 |
| Uzbekistan | Europe & Central Asia | 580 | 274 | 887 | 142 | 67 | 217 |
| Vanuatu | East Asia & Pacific | 661 | 310 | 1,013 | 651 | 305 | 998 |
| Vietnam | East Asia & Pacific | 630 | 308 | 951 | 194 | 95 | 292 |
| Zambia | Sub-Saharan Africa | 81 | 22 | 139 | 26 | 7 | 45 |
| Zimbabwe | Sub-Saharan Africa | 56 | 12 | 100 | 30 | 7 | 54 |
